# Cathepsins and Parkinson’s disease: insights from Mendelian randomization analyses

**DOI:** 10.3389/fnagi.2024.1380483

**Published:** 2024-06-05

**Authors:** Aishanjiang Yusufujiang, Shan Zeng, Hongyan Li

**Affiliations:** ^1^Department of Graduate School, Xinjiang Medical University, Ürümqi, China; ^2^Department of Neurology, People’s Hospital of Xinjiang Uygur Autonomous Region, Ürümqi, China; ^3^Xinjiang Clinical Research Center for Stroke and Neurological Rare Disease, Ürümqi, China

**Keywords:** Parkinson’s disease, cathepsin B, Mendelian randomization, neurodegeneration, biomarkers

## Abstract

**Background:**

Parkinson’s disease (PD), the second most prevalent neurodegenerative condition, has a multifaceted etiology. Cathepsin-cysteine proteases situated within lysosomes participate in a range of physiological and pathological processes, including the degradation of harmful proteins. Prior research has pointed towards a potential link between cathepsins and PD; however, the precise causal relationship between the cathepsin family and PD remains unclear.

**Methods:**

This study employed univariate and multivariate Mendelian randomization (MR) analyses to explore the causal relationship between the nine cathepsins and Parkinson’s disease (PD) risk. For the primary analysis, genome-wide association study (GWAS) summary statistics for the plasma levels of the nine cathepsins and PD was obtained from the INTERVAL study and the International Parkinson’s Disease Genomics Consortium. GWAS for PD replication analysis were obtained from the FinnGen consortium, and a meta-analysis was performed for the primary and replication analyses to evaluate the association between genetically predicted cathepsin plasma levels and PD risk. After identifying significant MR estimates, genetic co-localization analyses were conducted to determine whether shared or distinct causal variants influenced both cathepsins and PD.

**Results:**

Elevated cathepsin B levels were associated with a decreased risk of PD in univariate MR analysis (odds ratio [OR] = 0.890, 95% confidence interval [CI]: 0.831–0.954, pFDR = 0.009). However, there was no indication that PD affected cathepsin B levels (OR = 0.965, 95% CI: 0.858–1.087, *p* = 0.852). In addition, after adjusting for the remaining cathepsins, cathepsin B levels independently and significantly contributed to the reduced risk of PD in multivariate MR analysis (OR = 0.887, 95% CI: 0.823–0.957, *p* = 0.002). The results of the replication MR analysis with the FinnGen GWAS for PD (OR = 0.921, 95% CI: 0.860–0.987, *p* = 0.020) and meta-analysis (OR = 0.905, 95% CI: 0.862–0.951, *p* < 0.001) were consistent with those of the primary analysis. Colocalization analysis did not provide any evidence of a shared causal variant between cathepsins and PD (PP.H4.abf = 0.005).

**Conclusion:**

This genetic investigation supports the hypothesis that cathepsin B exerts a protective effect against PD. The quantification of cathepsin B levels could potentially serve as a predictive biomarker for susceptibility to PD, providing new insights into the pathomechanisms of the disease and possible interventions.

## Introduction

Parkinson’s disease (PD) is a neurodegenerative condition characterized by degeneration of dopaminergic neurons in the substantia nigra pars compacta. Disease progression is closely linked to the accumulation of alpha-synuclein and abnormal protein degradation, and proteases, particularly cathepsins, play a key role in the attenuation of pathological protein aggregates (Reiser et al., 2010; Rai et al., 2022; Stoka et al., 2023). Several studies have established an association between PD and cathepsin activity, indicating their possible role in the etiology of the disease (Yelamanchili et al., 2011; Pišlar et al., 2018; McGlinchey et al., 2019; Pal et al., 2019; Yuan et al., 2021; Milanowski et al., 2022; Stoka et al., 2023).

Proteases, including those of the cathepsin family, are lysosomal enzymes that are essential for maintaining cellular homeostasis. Cathepsins are cysteine proteases that belong to the papain superfamily. They are involved in various cellular processes such as autophagy, cell signaling, and protein and lipid turnover (Fonović et al., 2014). Owing to their diverse functions, they contribute to many diseases, including neurological conditions such as Parkinson’s disease (Stoka et al., 2023).

Experimental studies have consistently identified cathepsins as important contributors to PD pathogenesis. Although the study by Mantle et al. (1995) did not identify significant differences in cathepsin activity between PD patients and controls, other studies have identified an increase in the expression of cathepsin B, D, and X in animal models of PD (Pišlar et al., 2018; Gan et al., 2019). This finding suggests a possible association between disease initiation and development. Furthermore, degradation of the alpha-synuclein C-terminal, which is caused by cathepsin activity, has been observed in Lewy bodies. This is believed to be related to the formation of amyloid plaques and development of Parkinson’s disease (McGlinchey et al., 2019). Furthermore, interactions between cathepsins and other biomarkers, as well as genetic variabilities such as apolipoprotein E and Brain-Derived Neurotrophic Factor (BDNF), may contribute to the risk of Parkinson’s disease. This highlights the genetic complexity of the disease development (Schulte et al., 2003; Pal et al., 2019; Milanowski et al., 2022).

Advances in genomic science have strengthened our understanding of the role of heredity in disease development. Genetic variants from genome-wide association studies (GWAS) can be used as instrumental variables in Mendelian randomization (MR) studies to establish causal relationships between exposure and outcome. We conducted MR analyses in the context of PD in order to determine the causal effect of various cathepsins on the risk of developing PD (Emdin et al., 2017). In this study, univariate and multivariate MR techniques were employed to identify genetic level associations, and colocalization analyses were performed to examine shared genetic loci.

## Methods

### Data sources

**Figure 1:**
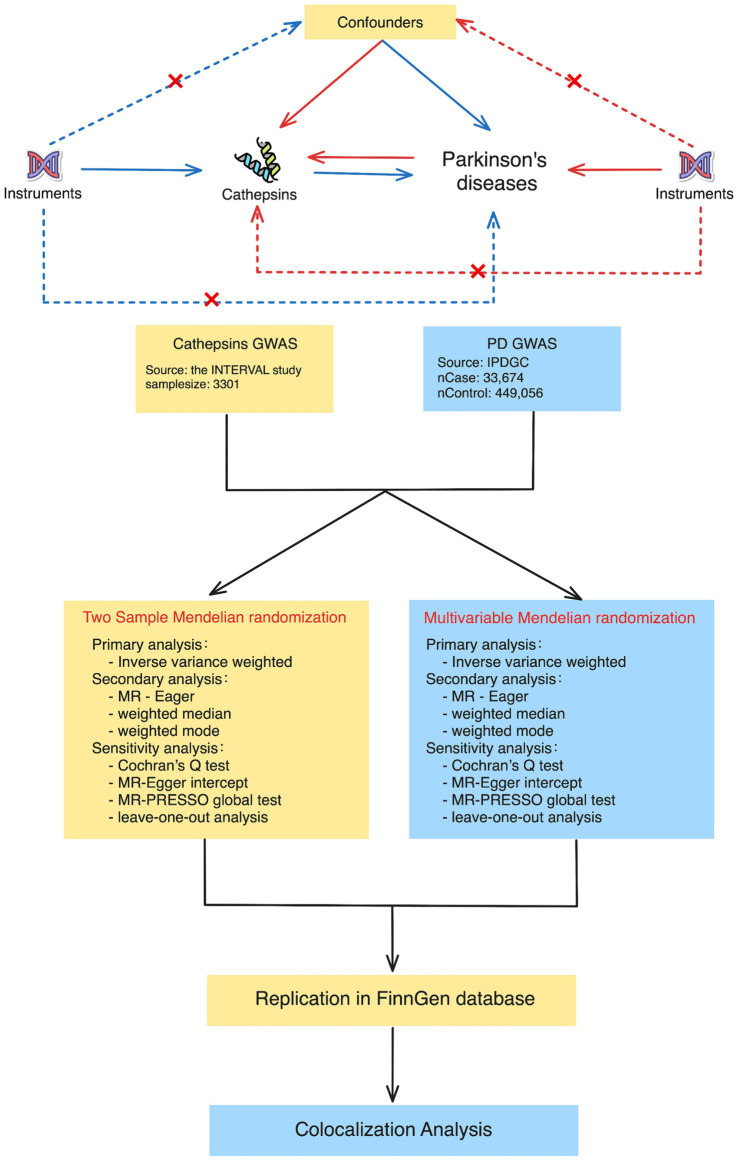
Study design for Mendelian randomization and colocalization analyses between cathepsins and Parkinson’s disease. The study approach contained two main phases: in the first phase, two-sample Mendelian randomization (on the left) and multivariable Mendelian randomization (on the right). The procedures encompass primary analysis techniques, such as inverse variance weighted, as well as secondary analysis techniques, such as MR-Egger and weighted medians. The sensitivity analyses included Cochran’s Q test, the MR-Egger intercept, the MR-PRESSO global test, and leave-one-out analysis. During the second phase, we conducted colocalization analyses to determine whether there was a shared genetic variant between positive cathepsin from the first phase and Parkinson’s disease.

This study used publicly accessible datasets. GWAS summary statistics for cathepsin levels were obtained from the INTERVAL study, which included 3,301 European participants (Sun et al., 2018). This study was approved by The National Research Ethics Service, and informed consent was obtained from all participants. PD GWAS data were obtained from the International Parkinson’s Disease Genomics Consortium, which consists of 33,674 PD cases and 449,056 controls (Nalls et al., 2019). To ensure the stability of the significant results, we extracted GWAS data on PD (4,681 cases and 407,500 controls) from the FinnGen Consortium Freeze 10 database for replication analysis (Kurki et al., 2023). The data sources and study flowchart are presented in Table 1 and Figure 1.

**Table 1:** Data sources for cathepsins and Parkinson’s disease.

Trait name	Data sources	Population	Samplesize	PMID	IEU Trait ID
Cathepsin B	The INTERVAL study	European	3,301	29,875,488	prot-a-718
Cathepsin E	The INTERVAL study	European	3,301	29,875,488	prot-a-720
Cathepsin F	The INTERVAL study	European	3,301	29,875,488	prot-a-722
Cathepsin G	The INTERVAL study	European	3,301	29,875,488	prot-a-723
Cathepsin H	The INTERVAL study	European	3,301	29,875,488	prot-a-725
Cathepsin O	The INTERVAL study	European	3,301	29,875,488	prot-a-726
Cathepsin S	The INTERVAL study	European	3,301	29,875,488	prot-a-727
Cathepsin L2	The INTERVAL study	European	3,301	29,875,488	prot-a-728
Cathepsin Z	The INTERVAL study	European	3,301	29,875,488	prot-a-729
Parkinson’s Disease	International Parkinson’s Disease Genomics Consortium	European	482,730	31,701,892	Ieu-b-7

### Instrument selection

The selection of Cathepsin-related instrumental variables (IVs) for this study was carried out meticulously, adhering to stringent criteria. These criteria included ensuring that the IVs exhibited low linkage disequilibrium (LD) with an r^2^ value below 0.001 within a 10,000 kb window and had *p*-values below 5 × 10^−6^. Similarly, for the reverse Mendelian randomization analysis related to PD, the same criteria were applied, with the p-value threshold set at 5 × 10^−8^. Single Nucleotide Polymorphisms (SNPs) in the exposure data can be found in Supplementary Tables S1, S2. The rigorous selection process involved identifying SNPs with genome-wide significance (*p* < 5 × 10^−6^) as potential instrumental variables, excluding SNPs associated with the outcome (*p* < 0.05), considering linkage disequilibrium through a clumping procedure, assessing and correcting for pleiotropy using the MR-PRESSO test, verifying instrument strength with the F-statistic, and filtering IVs based on exposure-outcome associations. These steps ensure the robustness and validity of the instrumental variables used in this study for accurate causal inference in Mendelian randomization analysis.

Considering that PD is susceptible to lifestyle, smoking, alcohol consumption, use of psychotropic drugs and Type 2 diabetes, we queried the SNPs of the above positive results using NHGRI-EBI Catalog database^1^ with therdhold of *p* = 5 × 10^−5^ and 2 SNPs (rs1260326 and rs34593439) in IVs of cathepsins associated with the above confounding factors (detailed in Supplementary Table S6).

### MR analysis

The inverse-variance weighted (IVW) method has been predominantly utilized in MR investigations to estimate effect size (Emdin et al., 2017). The Wald ratio in IVW was used to weigh the effect of each variant on exposure in relation to the risk of disease. A random-effects inverse variance meta-analysis was employed to merge the individual MR estimates. MR findings were validated using the MR Egger, Weighted Median, and Weighted Mode methods (Burgess et al., 2017). The robustness of assumptions and the presence of outliers and horizontal pleiotropy were evaluated using sensitivity analyses and statistical tests, such as Cochran’s Q test, MR-PRESSO global test, leave-one-out analysis, and MR-Egger intercept. The MR-PRESSO distortion test assesses distortions in causal estimates (Verbanck et al., 2018).

To further evaluate the independent effects of cathepsins, additional multivariate Mendelian randomization was used to investigate whether the impact of each individual cathepsin was dependent on other cathepsins. This study employed multivariable MR to assess the direct causal impact of several cathepsins on the risk of PD in a single analysis using the Mendelian randomization package (Yavorska et al., 2017).

### Replication analysis meta analysis

To validate the robustness of the results, the FinnGen GWAS database was used as a second independent consortium for data on Parkinson’s (Kurki et al., 2023). We conducted a replicated MR analysis for significant results, and a meta-analysis to explore the combined effects.

### Colocalization analysis

To identify whether cathepsins genetically linked with PD share a causal variant, we conducted colocalization analysis. Bayesian testing was used to conduct colocalization analysis, utilizing the minor allele frequency (MAF) for approximations (Giambartolomei et al., 2014). We used the coloc.abf function to examine the genetic regions around the Cathepsin B gene, specifically focusing on a 50 kb window centered on the gene’s location on chromosome 8. For each pair of traits, we examined five hypotheses: H0 (no SNP causing the traits), H1 (associated with trait 1), H2 (association with trait 2), H3 (two separate SNPs causing the traits independently), and H4 (one SNP causing both traits). Colocalization was considered to have occurred when the posterior probability (SNP.PP.H4) was greater than 0.8. The R package Coloc was used in this study.

### Statistical analysis

All statistical analyses were performed by using the “TwoSampleMR” (Hemani et al., 2018), “MR-PRESSO” (Verbanck et al., 2018), “MendelianRandomization (Yavorska et al., 2017),” “coloc” (Rasooly et al., 2022) and “forestploter” packages (Zheng et al., 2020) in R (version 4.2.1.).

## Results

### Forward univariable MR analysis

In the forward univariate MR analysis, we investigated the impact of nine cathepsins (B, E, F, G, H, L2, O, S, and Z) on the risk of PD. The analysis employed multiple MR methods, including Inverse Variance Weighted (IVW), MR Egger, Weighted Median, and Weighted Mode, using 9–22 single nucleotide polymorphisms as instrumental variables.

**Figure 2:**
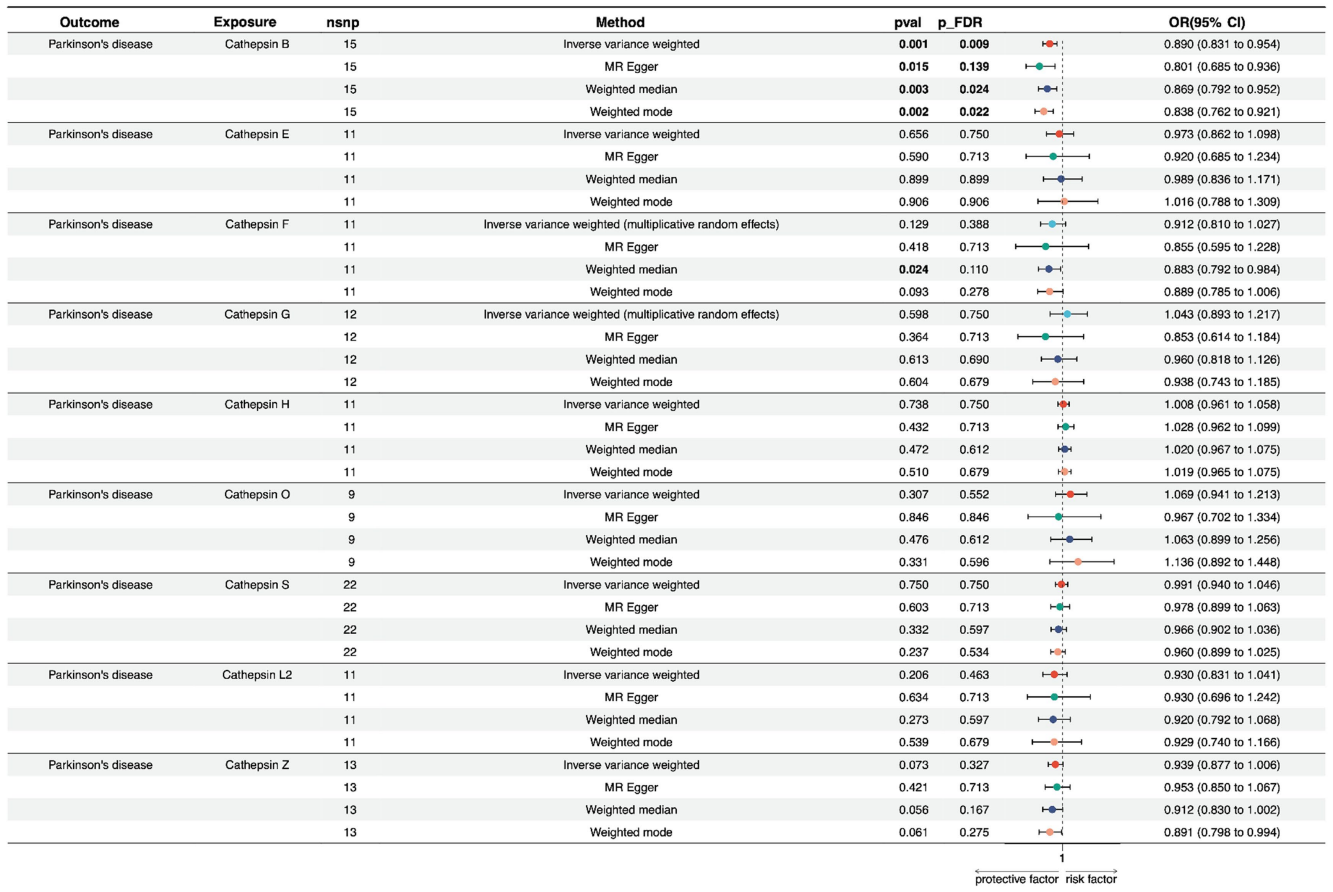
Forest plots of univariable Mendelian randomization analysis of the relationship between various Cathepsins on Parkinson’s disease.

The results showed that Cathepsin B exposure was associated with a decreased risk of PD across all the MR methods. Specifically, the IVW method showed an odds ratio (OR) of 0.890 (95% CI: 0.831–0.954), and the result was statistically significant after multiple testing corrections (p_FDR = 0.009). The MR Egger, Weighted Median, and Weighted Mode methods also supported this finding, with consistent directions of effects and significance levels. For cathepsin E, F, G, H, O, S, Z, and, and L2, none of the MR methods indicated a significant relationship with PD risk, with *p*-values exceeding the conventional threshold of 0.05, and odds ratios close to null. The heterogeneity tests (Q_pval) were mostly non-significant, suggesting that the effect estimates were consistent across the different genetic instruments. The MR-Egger intercept and PRESSO did not indicate the presence of directional pleiotropy or outliers, confirming the robustness of our findings (Figure 2; Supplementary Table S3).

To address potential pleiotropic bias arising from trans-pQTLs, we conducted a univariate Mendelian Randomization (MR) analysis using exclusively the cis-pQTLs for each Cathepsin protein. Specifically, we included rs1692819 for Cathepsin B, rs1791679 for Cathepsin F, rs62013235 for Cathepsin H, and rs41271951 for Cathepsin S as the sole cis-pQTLs in our MR analysis, employing the Wald Ratio method. The results revealed that utilizing rs1692819 as the cis-pQTL for Cathepsin B showed a significant association with an odds ratio (OR) of 0.829 (95% CI: 0.752–0.915, p_FDR < 0.001). However, no significant associations were found for Cathepsin F (OR = 0.897, 95% CI: 0.769–1.046, p_FDR = 0.332), Cathepsin H (OR = 1.044, 95% CI: 0.888–1.228, p_FDR = 0.600), and Cathepsin S (OR = 0.965, 95% CI: 0.898–1.037, p_FDR = 0.446) with PD. This result reaffirmed the stability and reliability of our results, corroborating the initial findings of our study (Supplementary Figure S2).

### Reverse univariable MR analysis

We conducted a reverse MR analysis to explore the potential causal effect of PD on the expression levels of various cathepsins. Multiple MR methods were employed, including Inverse Variance Weighted (IVW), MR Egger, Weighted Median, and Weighted Mode, using 5–11 single nucleotide polymorphisms as instrumental variables.

**Figure 3:**
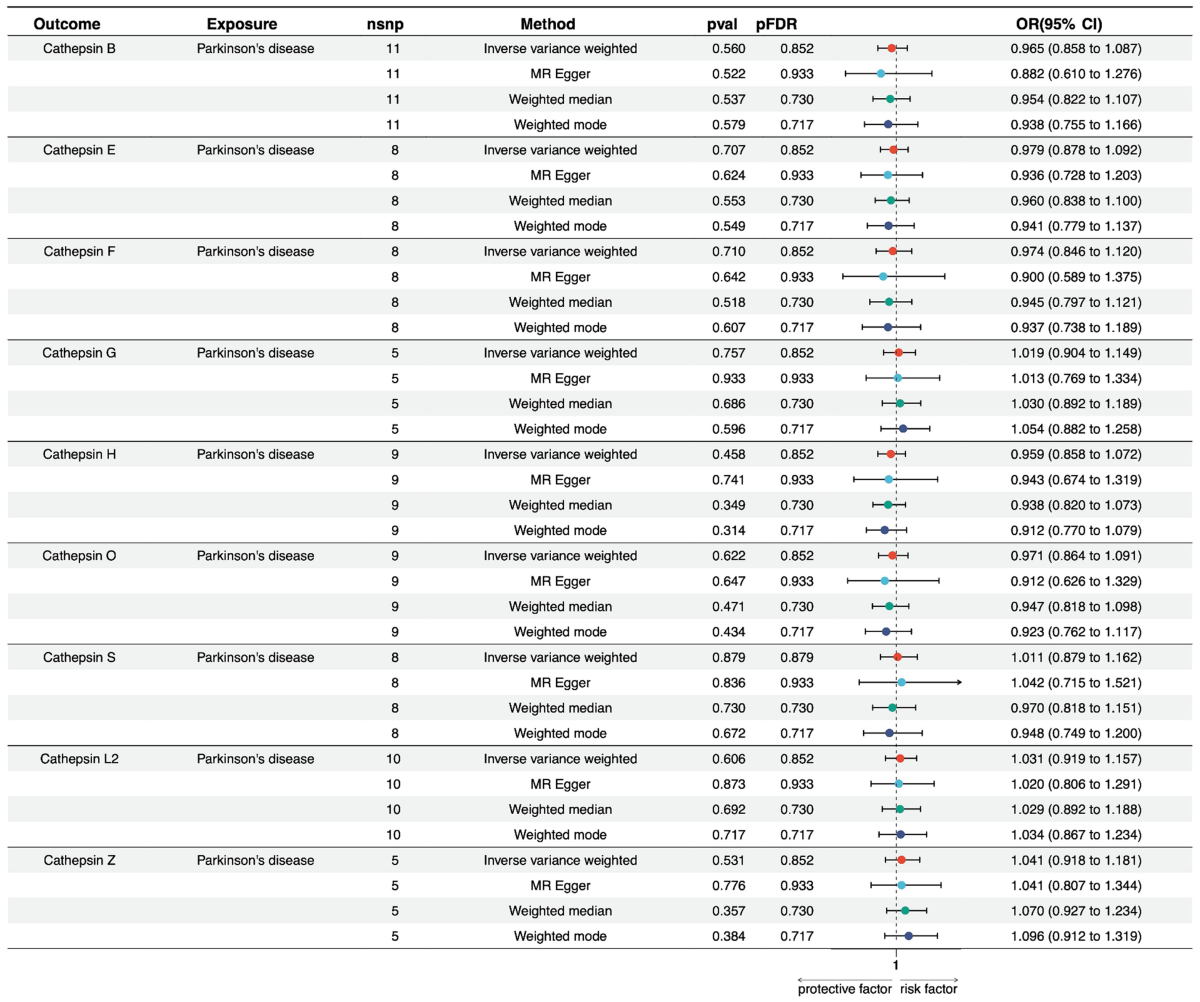
Forest plots of univariable Mendelian randomization analysis of the relationship between Parkinson’s disease and various cathepsins.

Regarding PD and Cathepsin B levels, none of the MR methods showed a significant effect on PD expression. The IVW method yielded a beta coefficient (b) of −0.035 (standard error [SE] = 0.060, *p* = 0.560), indicating a non-significant effect of PD on Cathepsin B levels. The MR Egger, Weighted Median, and Weighted Mode methods all supported these findings, with *p*-values exceeding the threshold for statistical significance. Furthermore, for PD on cathepsin E, F, G, H, O, S, L2, and Z levels, non-significant results suggest that within the power of our analysis, PD does not have a detectable causal effect on cathepsin expression levels (Figure 3; Supplementary Table S4).

### Multivariable MR analysis

**Figure 4:**
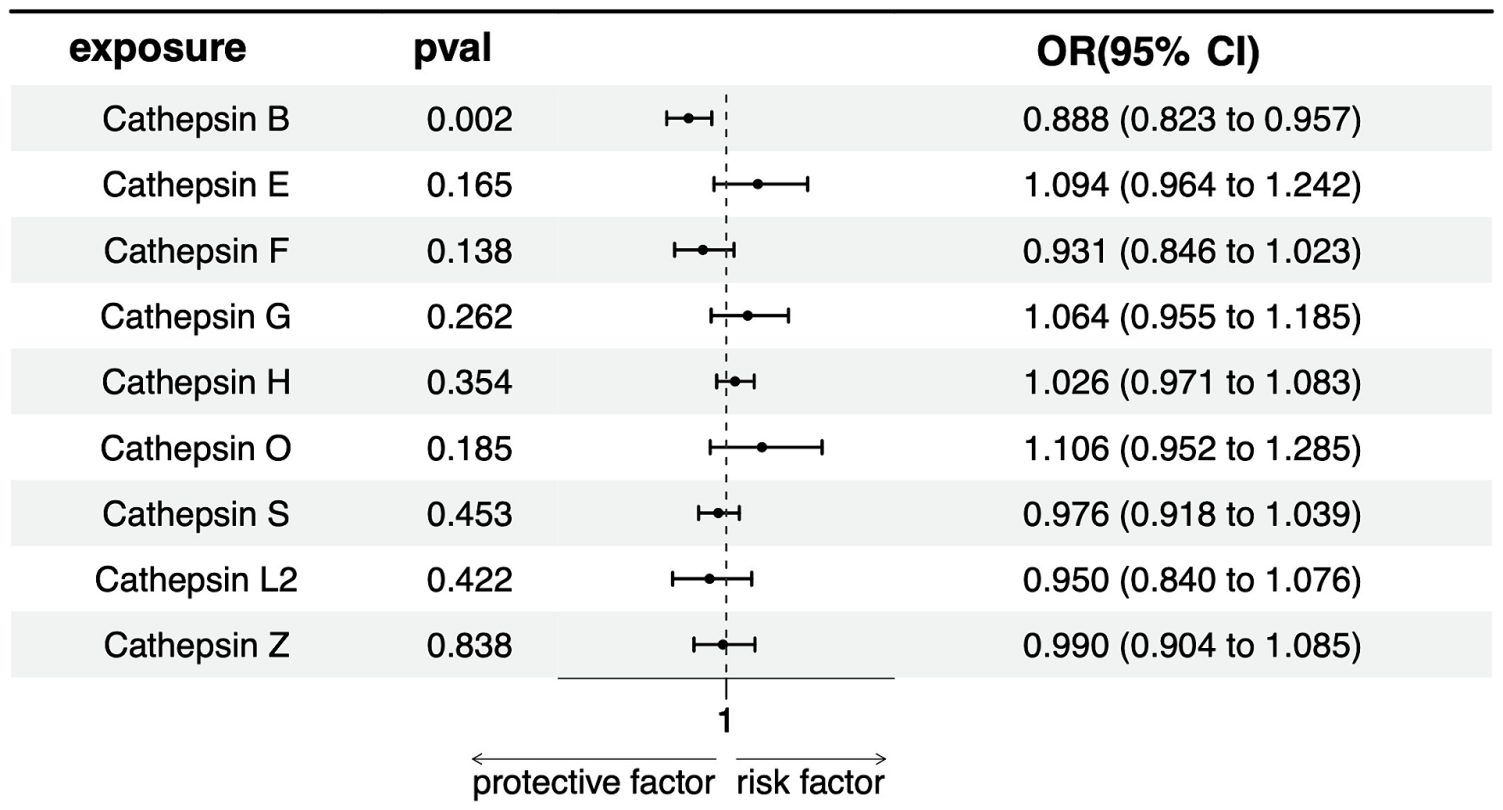
Forest plots of multivariable Mendelian randomization analysis of the relationship between various Cathepsins on Parkinson’s disease.

In our multivariate Mendelian Randomization (MR) analysis evaluating the influence of different cathepsins as exposures on PD, following the screening of 9 cathepsins using a rigorous threshold of *p* = 5 × 10^−6^, r^2^ = 0.001, and kb = 10,000, subsequent refinement procedures encompassing deduplication, clumping, and harmonization culminated in the identification of 10 SNPs as IVs for the MVMR analysis. The results show that only cathepsin B showed a significant negative association with PD risk (OR = 0.887, 95% CI = 0.823–0.957, *p* = 0.002), indicating a potential protective effect against PD. None of the other cathepsins (E, F, G, H, O, S, L2, Z) was significantly associated with PD, with *p*-values exceeding the threshold for significance (Figure 4). The lack of significant associations for these cathepsins suggests that they might not be causally related to PD, at least within the scope of this analysis.

### Replication and meta-analysis

**Figure 5:**
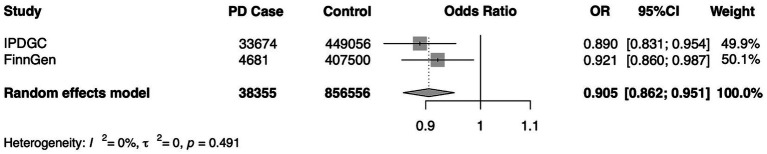
A meta-analysis of the causal association of cathepsin B and Parkinson’s Disease. IPDGC, International Parkinson’s Disease Genomics Consortium; FinnGen, the FinnGen consortium; OR, odds ratio; CI, confidence interval.

To verify the stability of the results, another independent FinnGen database was used for repeated MR analysis, and a further meta-analysis was performed. Replicated MR analysis between Cathepsin B and PD showed a similar effect in the FinnGen consortium (OR = 0.921, 95% CI = 0.860–0.987, *p* = 0.020 for the IVW method) (Table 2) and remained significant in the combined meta-analysis (OR = 0.905, 95% CI = 0.862–0.951, *p* < 0.0001) (Figure 5).

**Table 2:** Replication MR analysis cathepsin B on Parkinson’s disease in FinnGen consortium.

Outcome	Exposure	Method	nsnp	OR	LCI 95%	UCI 95%	pval
Parkinson’s disease	Cathepsin B	Inverse variance weighted	19	0.921	0.860	0.987	0.020
Parkinson’s disease	Cathepsin B	MR Egger	19	0.950	0.807	1.118	0.545
Parkinson’s disease	Cathepsin B	Weighted median	19	0.931	0.835	1.039	0.201
Parkinson’s disease	Cathepsin B	Weighted mode	19	0.928	0.824	1.044	0.229

nsnp, number of SNP; LCI, lower confidence interval; UCI, upper confidence interval; pval, *p* value.

### Colocalization analysis

Colocalization analysis was used to detect genetic variants shared between cathepsin B and PD. Therefore, we did not find any substantial evidence of a shared causal variant influencing either cathepsin B levels or PD (PP.H4.abf = 0.005) (Supplementary Figure S1; Supplementary Table S5).

## Discussion

This study aimed to investigate the complex association between Cathepsins and PD using Mendelian randomization and colocalization approaches. Our findings are the first attempt in the field of PD pathogenesis, shedding light on the potential protective role of cathepsin B, an enzyme integral to the degradation of pathological proteins such as alpha-synuclein.

Using univariate and multivariate MR analyses, our study provides compelling evidence that cathepsin B levels are negatively correlated with susceptibility to PD. A variety of sensitivity and replication analyses have provided further support for this association, thereby enhancing the consistency and dependability of our findings. Notably, our data show that cathepsin B and PD do not share any genetic variations, indicating a complex interaction that requires further research.

At the molecular level, the protective mechanisms of cathepsin B against PD may involve its crucial function in the degradation of alpha-synuclein through autophagy, a critical process that prevents the harmful accumulation of this protein. The results of Jones-Tabah et al. (2023) and McGlinchey et al. (2019) corresponded with our findings, highlighting the crucial role of cathepsin B in maintaining lysosomal function and preventing the development of neurotoxic aggregates.

To expand our understanding of the possible consequences of cathepsin B activity, Bai et al. (2018) and Nakanishi (2020) investigated the effect of this enzyme on oxidative stress and neuroinflammation, which are known to contribute to PD and other neurodegenerative disorders. Moreover, research conducted by Kim et al. (2022) and Almeida et al. (2020) indicated that focusing on the lysosomal pathway, specifically cathepsin B, could potentially become a highly effective therapeutic approach not only for PD but also for various other neurodegenerative disorders.

By contrast, Tsujimura et al. (2015) offer an alternative viewpoint by suggesting that cathepsin B could potentially facilitate the development of intracellular alpha-synuclein aggregates, which are characteristic features of PD pathology. The apparent contradiction in cathepsin B function underscores the enzyme’s intricate and situationally dependent roles in cellular processes, which may differ during the distinct phases of the disease.

However, our study had some limitations. Firstly, the homogeneity of our sample population, which primarily consisted of individuals of European ancestry, may have limited the generalizability of our results. To enhance the generalizability of our findings and firmly establish cathepsin B as a feasible biomarker for PD, future research should incorporate a more heterogeneous sample. Secondly, Our study not observing colocalization between Cathepsin B and PD, indicating a potential confounding effect of linkage disequilibrium between the cathepsin B pQTL and PD risk-associated variants. This observation aligns with findings from Zheng et al. (2020), underscoring the likelihood that the significant associations identified through MR analysis may be influenced by LD rather than reflecting a direct causal relationship. The absence of colocalization emphasizes the complexity inherent in interpreting MR results in the context of potential LD confounders, shedding light on the nuanced interplay between genetic factors and disease susceptibility in PD. lastly, The consideration of tissue specificity in protein biomarker analysis emerges as a pertinent limitation in our study, with plasma serving as the primary source of protein measurements despite the relevance of brain tissue for PD research. This discrepancy underscores a key aspect highlighted in the work by Yang et al. (2021), wherein distinct tissue-specific protein quantitative trait loci (pQTLs) profiles are reported. The observation that different tissues exhibit unique pQTL effects emphasizes the importance of utilizing brain-related tissues for PD studies to capture more relevant and context-specific insights. Our reliance on plasma-derived data, while informative, introduces a limitation in the interpretation of our findings, as the tissue-specificity of pQTLs may not be fully captured in this context.

In summary, our MR analysis provides significant data supporting the neuroprotective effect of cathepsin B and its potential as a therapeutic target in PD. The findings of this study strongly encourage additional investigations into the biological roles of cathepsin B and its possible use as an early biomarker for diagnosing PD. Subsequent investigations should focus on overcoming the recognized constraints and deepening our understanding to effectively utilize the therapeutic potential of cathepsin B in combating PD and other neurodegenerative disorders.

## Data availability statement

The main statistical analyses were conducted using TwoSampleMR (v.0.5.7), MR-PRESSO(v1.0), MendelianRandomization(v0.90), and coloc(v5.2.3) in R software (version 4.1.2). The original datasets of the current study are available from the IEU Open Gwas Project (https://gwas.mrcieu.ac.uk/) and FinnGen GWAS database (https://www.finngen.fi/fi).

## Ethics statement

This study utilized summary data from public GWAS datasets and the original study had already obtained ethical approval, no additional ethical approval was required. The research was conducted in accordance with local legislation and institutional requirements. Written informed consent from participants or their legal guardians/next of kin was not necessary, as this study only used summary data from public datasets and ethical approval had been obtained in the original study.

## Author contributions

AY: Conceptualization, Formal analysis, Methodology, Writing – original draft. SZ: Data curation, Methodology, Writing – review & editing. HL: Conceptualization, Data curation, Supervision, Writing – review & editing.

## Footnotes

^1^ https://www.ebi.ac.uk/gwas/

## References

[ref1] Almeida M. F. Bahr B. A. Kinsey S. T. (2020). Endosomal-lysosomal dysfunction in metabolic diseases and Alzheimer’s disease. Int. Rev. Neurobiol. 154, 303–324. doi: 10.1016/bs.irn.2020.02.012, PMID: 32739009 PMC8428780

[ref2] Bai H. Yang B. Wenfeng Y. Xiao Y. Dejun Y. Zhang Q. (2018). Cathepsin B links oxidative stress to the activation of NLRP3 Inflammasome. Exp. Cell Res. 362, 180–187. doi: 10.1016/j.yexcr.2017.11.015, PMID: 29196167

[ref9001] Burgess S. Thompson S. G. (2017). Interpreting findings from mendelian randomization using the MR-Egger method. Eur J Epidemiol. 32, 377–389. doi: 10.1007/s10654-017-0255-x 28527048 PMC5506233

[ref3] Emdin C. Amit V. K. Kathiresan S. (2017). Mendelian Randomization. JAMA 318:1925. doi: 10.1001/jama.2017.17219 29164242

[ref4] Fonović M. Turk B. Fonović M. Turk B. Fonović M. Turk B. (2014). Cysteine Cathepsins and extracellular matrix degradation. Biochim. Biophys. Acta, Gen. Subj. 1840, 2560–2570. doi: 10.1016/j.bbagen.2014.03.017 24680817

[ref5] Gan P. Xia Q. Hang G. Zhou Y. Qian X. Wang X.. (2019). Knockdown of Cathepsin D protects dopaminergic neurons against Neuroinflammation-mediated neurotoxicity through inhibition of NF- κ B Signalling pathway in Parkinson’s disease model. Clin. Exp. Pharma. Physio. 46, 337–349. doi: 10.1111/1440-1681.13052 30485484

[ref6] Giambartolomei C. Vukcevic D. Schadt E. E. Franke L. Hingorani A. D. Wallace C.. (2014). Bayesian test for Colocalisation between pairs of genetic association studies using summary statistics. PLoS Genet. 10:e1004383. doi: 10.1371/journal.pgen.1004383, PMID: 24830394 PMC4022491

[ref9003] Hemani G. Zheng J. Elsworth B. Wade K. H. Haberland V. Baird D. (2018). The MR-Base platform supports systematic causal inference across the human phenome. Elife. 7:e34408. doi: 10.7554/eLife.34408 29846171 PMC5976434

[ref7] Jones-Tabah J. He K. Senkevich K. Karpilovsky N. Deyab G. Cousineau Y.. (2023). The Parkinson’s disease risk gene Cathepsin B promotes Fibrillar alpha-Synuclein clearance, lysosomal function and Glucocerebrosidase activity in dopaminergic neurons. bioRxiv. doi: 10.1101/2023.11.11.566693 PMC11587650 39587654

[ref8] Kim M. J. Jeong H. Krainc D. (2022). Lysosomal ceramides regulate Cathepsin B-mediated processing of Saposin C and Glucocerebrosidase activity. Hum. Mol. Genet. 31, 2424–2437. doi: 10.1093/hmg/ddac047, PMID: 35181782 PMC9307309

[ref9] Kurki M. I. Karjalainen J. Palta P. Sipilä T. P. Kristiansson K. Donner K. M.. (2023). FinnGen provides genetic insights from a well-phenotyped isolated population. Nature 613, 508–518. doi: 10.1038/s41586-022-05473-8, PMID: 36653562 PMC9849126

[ref10] Mantle D. Falkous G. Ishiura S. Perry R. H. Perry E. K. Mantle D.. (1995). Comparison of Cathepsin protease activities in brain tissue from Normal cases and cases with Alzheimer’s disease, Lewy body dementia, Parkinson’s disease and Huntington’s disease. J. Neurol. Sci. 131, 65–70. doi: 10.1016/0022-510X(95)00035-Z, PMID: 7561949

[ref11] McGlinchey R. P. Lacy S. M. Huffer K. E. Tayebi N. Sidransky E. Lee J. C.. (2019). C-terminal α-synuclein truncations are linked to cysteine cathepsin activity in Parkinson's disease. J. Biol. Chem. 294, 9973–9984. doi: 10.1074/jbc.RA119.008930, PMID: 31092553 PMC6597809

[ref12] Milanowski L. M. Hou X. Bredenberg J. M. Fiesel F. C. Cocker L. T. Soto-Beasley A. I.. (2022). Cathepsin B p.Gly284Val variant in Parkinson’s disease pathogenesis. Int. J. Mol. Sci. 23:7086. doi: 10.3390/ijms23137086, PMID: 35806091 PMC9266886

[ref13] Nakanishi H. (2020). Microglial Cathepsin B as a key driver of inflammatory brain diseases and brain aging. Neural Regen. Res. 15, 25–29. doi: 10.4103/1673-5374.264444, PMID: 31535638 PMC6862407

[ref14] Nalls M. A. Blauwendraat C. Vallerga C. L. Heilbron K. Bandres-Ciga S. Chang D.. (2019). Identification of novel risk loci, causal insights, and heritable risk for Parkinson’s disease: A Meta-analysis of genome-wide association studies. Lancet Neurol. 18, 1091–1102. doi: 10.1016/S1474-4422(19)30320-5, PMID: 31701892 PMC8422160

[ref15] Pal P. Sadhukhan T. Chakraborty S. Sadhukhan S. Biswas A. Das S. K.. (2019). Role of apolipoprotein E, Cathepsin D, and brain-derived neurotrophic factor in Parkinson’s disease: A study from eastern India. NeuroMolecular Med. 21, 287–294. doi: 10.1007/s12017-019-08548-4, PMID: 31134487

[ref16] Pišlar A. Tratnjek L. Glavan G. Živin M. Kos J. (2018). Upregulation of cysteine protease Cathepsin X in the 6-Hydroxydopamine model of Parkinson’s disease. Front. Mol. Neurosci. 11:412. doi: 10.3389/fnmol.2018.00412, PMID: 30450037 PMC6225071

[ref17] Rai M. Curley M. Coleman Z. Demontis F. Rai M. Curley M.. (2022). Contribution of proteases to the hallmarks of aging and to age-related neurodegeneration. Aging Cell 21:e13603. doi: 10.1111/acel.13603, PMID: 35349763 PMC9124314

[ref18] Rasooly D. Peloso G. M. Giambartolomei C. (2022). Bayesian genetic Colocalization test of two traits using coloc. Curr Protoc. 2:e627. doi: 10.1002/cpz1.627, PMID: 36515558

[ref19] Reiser J. Adair B. Reinheckel T. (2010). Specialized roles for cysteine Cathepsins in health and disease. J. Clin. Invest. 120, 3421–3431. doi: 10.1172/JCI42918, PMID: 20921628 PMC2947230

[ref20] Schulte T. Böhringer S. Schöls L. Müller T. Fischer C. Riess O.. (2003). Modulation of disease risk according to a Cathepsin D / apolipoprotein E genotype in Parkinson’s disease. J. Neural Transm. 110, 749–755. doi: 10.1007/s00702-003-0832-x, PMID: 12811635

[ref21] Stoka V. Vasiljeva O. Nakanishi H. Turk V. Stoka V. Vasiljeva O.. (2023). The role of cysteine protease Cathepsins B, H, C, and X/Z in neurodegenerative diseases and Cancer. IJMS 24:15613. doi: 10.3390/ijms242115613, PMID: 37958596 PMC10650516

[ref22] Sun B. B. Maranville J. C. Peters J. E. Stacey D. Staley J. R. Blackshaw J.. (2018). Genomic atlas of the human plasma proteome. Nature 558, 73–79. doi: 10.1038/s41586-018-0175-2, PMID: 29875488 PMC6697541

[ref23] Tsujimura A. Taguchi K. Watanabe Y. Tatebe H. Tokuda T. Mizuno T.. (2015). Lysosomal enzyme Cathepsin B enhances the aggregate forming activity of exogenous α -Synuclein fibrils. Neurobiol. Dis. 73, 244–253. doi: 10.1016/j.nbd.2014.10.011 25466281

[ref24] Verbanck M. Chen C.-Y. Neale B. Do R. (2018). Detection of widespread horizontal pleiotropy in causal relationships inferred from Mendelian randomization between complex traits and diseases. Nat. Genet. 50, 693–698. doi: 10.1038/s41588-018-0099-7, PMID: 29686387 PMC6083837

[ref25] Yang C. Farias F. H. G. Ibanez L. Suhy A. Sadler B. Fernandez M. V.. (2021). Genomic atlas of the proteome from brain, CSF and plasma prioritizes proteins implicated in neurological disorders. Nat. Neurosci. 24, 1302–1312. doi: 10.1038/s41593-021-00886-6, PMID: 34239129 PMC8521603

[ref26] Yavorska O. O. Burgess S. Yavorska O. O. Burgess S. Yavorska O. O. Burgess S.. (2017). MendelianRandomization: an R package for performing Mendelian randomization analyses using summarized data. Int. J. Epidemiol. 46, 1734–1739. doi: 10.1093/ije/dyx034, PMID: 28398548 PMC5510723

[ref27] Yelamanchili S. V. Chaudhuri A. D. Flynn C. T. Fox H. S. Yelamanchili S. V. Chaudhuri A. D.. (2011). Upregulation of Cathepsin D in the caudate nucleus of Primates with experimental parkinsonism. Mol. Neurodegener. 6:52. doi: 10.1186/1750-1326-6-52, PMID: 21777416 PMC3160400

[ref28] Yuan S. Mason A. M. Carter P. Burgess S. Larsson S. C. (2021). Homocysteine, B vitamins, and cardiovascular disease: A Mendelian randomization study. BMC Med. 19:97. doi: 10.1186/s12916-021-01977-8, PMID: 33888102 PMC8063383

[ref29] Zheng J. Haberland V. Baird D. Walker V. Haycock P. C. Hurle M. R.. (2020). Phenome-wide Mendelian randomization mapping the influence of the plasma proteome on complex diseases. Nat. Genet. 52, 1122–1131. doi: 10.1038/s41588-020-0682-6, PMID: 32895551 PMC7610464

