# Supplementary material for: Cathepsins and Parkinson’s disease: insights from Mendelian randomization analyses
Source: Front Aging Neurosci. 2024 Jun 5;16:1380483. doi: 10.3389/fnagi.2024.1380483 (PMC11188310; doi:10.3389/fnagi.2024.1380483)
Supplement: Supplementary file 1 [file Image_1.PDF]

Cathepsins	cis-pQTL	Method	P-FDR	OR(95% CI)
Cathepsin B	rs1692819	Wald ratio	**< 0.001**	**0.829 (0.752 to 0.915)**
Cathepsin F	rs1791679	Wald ratio	0.166	0.897 (0.769 to 1.046)
Cathepsin H	rs62013235	Wald ratio	0.600	1.044 (0.888 to 1.228)
Cathepsin S	rs41271951	Wald ratio	0.334	0.965 (0.898 to 1.037)

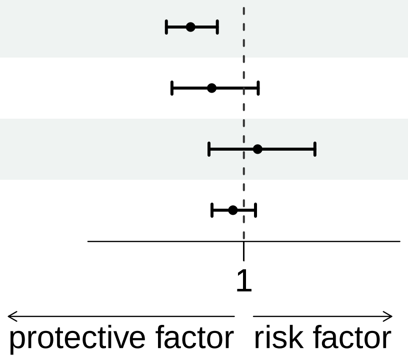

P<0.05 was considered statistically significant
