# Supplementary figures and images for: Cathepsins and Parkinson’s disease: insights from Mendelian randomization analyses
Source: Front Aging Neurosci. 2024 Jun 5;16:1380483. doi: 10.3389/fnagi.2024.1380483 (PMC11188310; doi:10.3389/fnagi.2024.1380483)

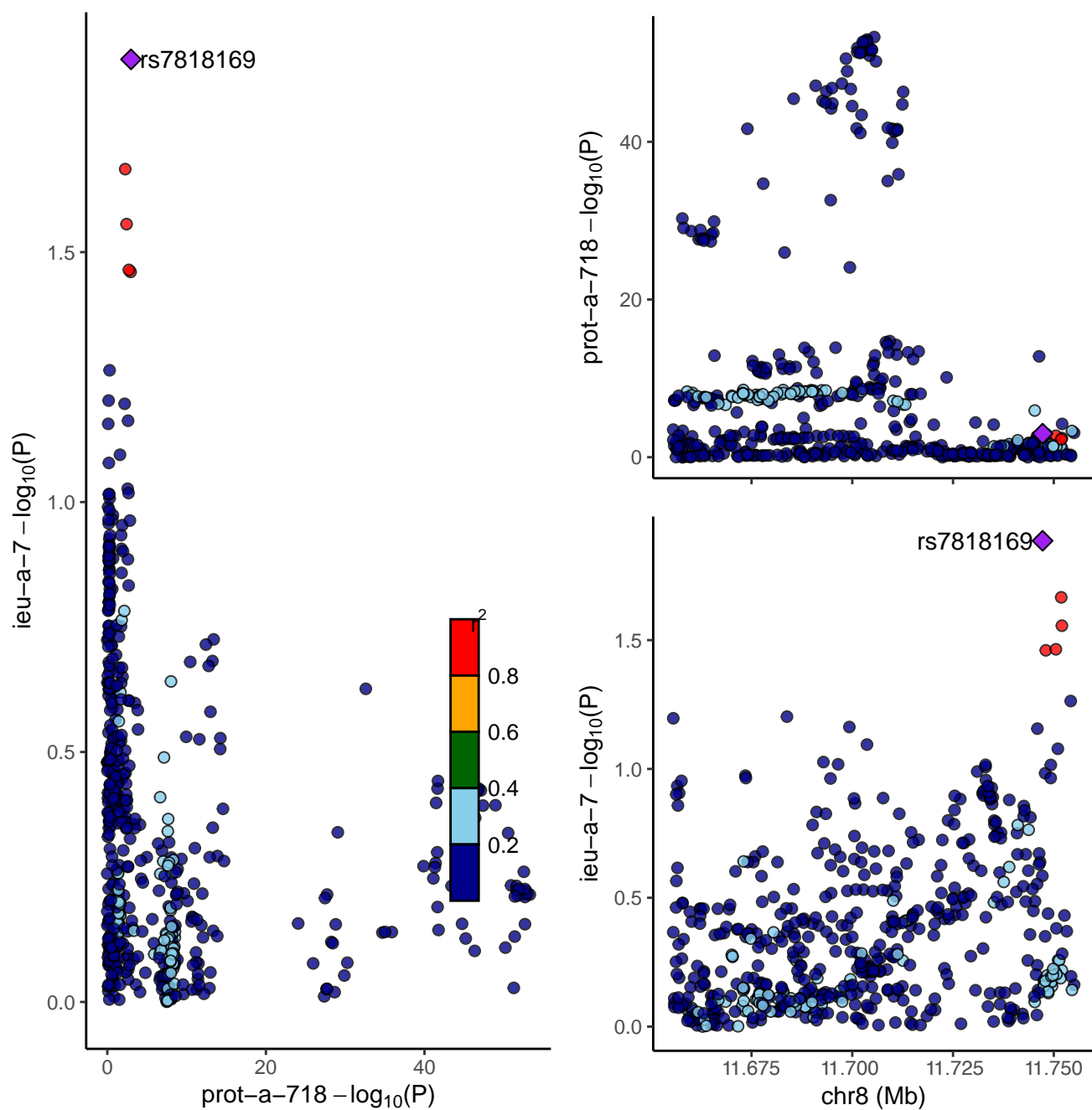

Supplement: Supplementary file 2 [file Image_2.PDF]
