# Supplementary material for: Cathepsins and Parkinson’s disease: insights from Mendelian randomization analyses
Source: Front Aging Neurosci. 2024 Jun 5;16:1380483. doi: 10.3389/fnagi.2024.1380483 (PMC11188310; doi:10.3389/fnagi.2024.1380483)
Supplement: Supplementary file 3 [file Table_1.DOCX]

Supplementary Material

**
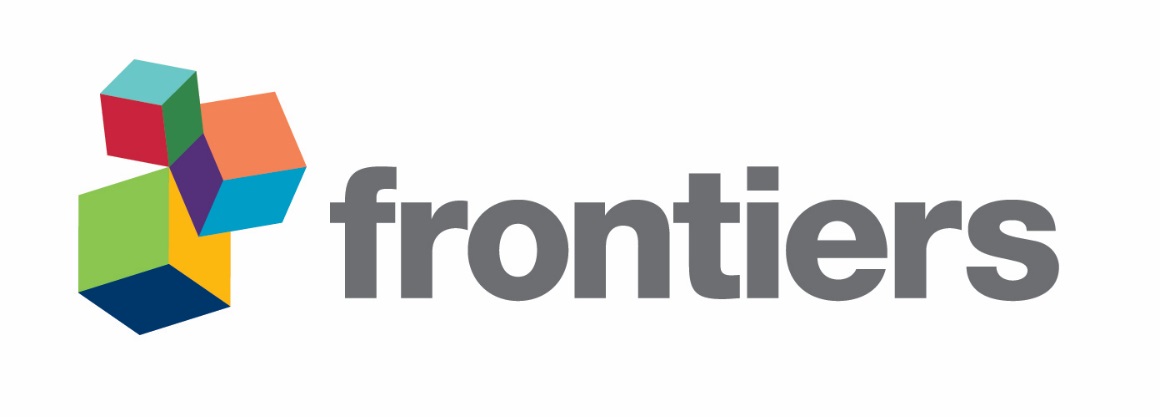
**

**Supplementary Figure 1.** Colocalization analysis with Manhattan plot and regional plot for cathepsin B and Parkinson's Disease

**Supplementary Figure 2.** Forest plot of univariable Mendelian randomization analysis of the relationship between various Cathepsins with cis-pQTL and Parkinson's disease.

**Supplementary Table 1.** Instrumental Variables for forward Mendelian randomization analysis between various Cathepsins and Parkinson's Disease

**Supplementary Table 2.** Instrumental Variables for reverse Mendelian randomization analysis between various Cathepsins and Parkinson's Disease

**Supplementary Table 3.** Detailed result for forward Mendelian randomization analysis between various Cathepsins and Parkinson's Disease

**Supplementary Table 4.** Detailed result for reverse Mendelian randomization analysis between various Cathepsins and Parkinson's Disease

**Supplementary Table 5.** Colocalization analysis result for Cathepsins B and Parkinson's Disease

**Supplementary Table 6.** Confounders screening result of Instrumental Variables for Cathepsins as Exposure
